# Intermolecular Chirality Modulation of Binaphthalene-Bridged Bisporphyrins With Chiral Diamines

**DOI:** 10.3389/fchem.2020.611257

**Published:** 2021-02-12

**Authors:** Wenxin Lu, Lei Gong, Chaorui Su, Qibao Wang, Qing Ling, Peng Wang, Dongdong Qi, Yongzhong Bian

**Affiliations:** ^1^ College of Chemical and Biological Engineering, Shandong University of Science and Technology, Qingdao, 266590, China; ^2^ Beijing Key Laboratory for Science and Application of Functional Molecular and Crystalline Materials, Department of Chemistry, University of Science and Technology Beijing, Beijing, China

**Keywords:** porphyrin, diamine, chirality, fluorescence spectroscopy, CD spectroscopy, chiral optical sensor

## Abstract

A new pair of 2,2ʹ-diamino-1,1ʹ-binaphthyl linked porphyrin dimers, (*R*)-/(*S*)-**H**, were synthesized to study their supramolecular interactions with a pair of chiral diamines ((*R*)-/(*S*)-PPDA) by using UV-Vis absorption, fluorescence and NMR titrations. The spectroscopic titrations indicated that sandwich-type 1:1 complexes were formed at low guest concentration and then transformed to 1:2 open complexes at high guest concentration. The supramolecular interactions afforded sensitive circular dichroism responses, and the CD signs of the 1:1 complexes are decided by the stereostructure of chiral diamine guests. Moreover, due to the shortened linking units, (*R*)-/(*S*)-**H** show more sensitive and predicable CD response than the previously reported hosts (*R*)-/(*S*)-**H1** and this can be reasonably explained by DFT molecular modeling. The present results suggest (*R*)-/(*S*)-**H** are promising for chiral optical sensing.

**GRAPHICAL ABSTRACT:**
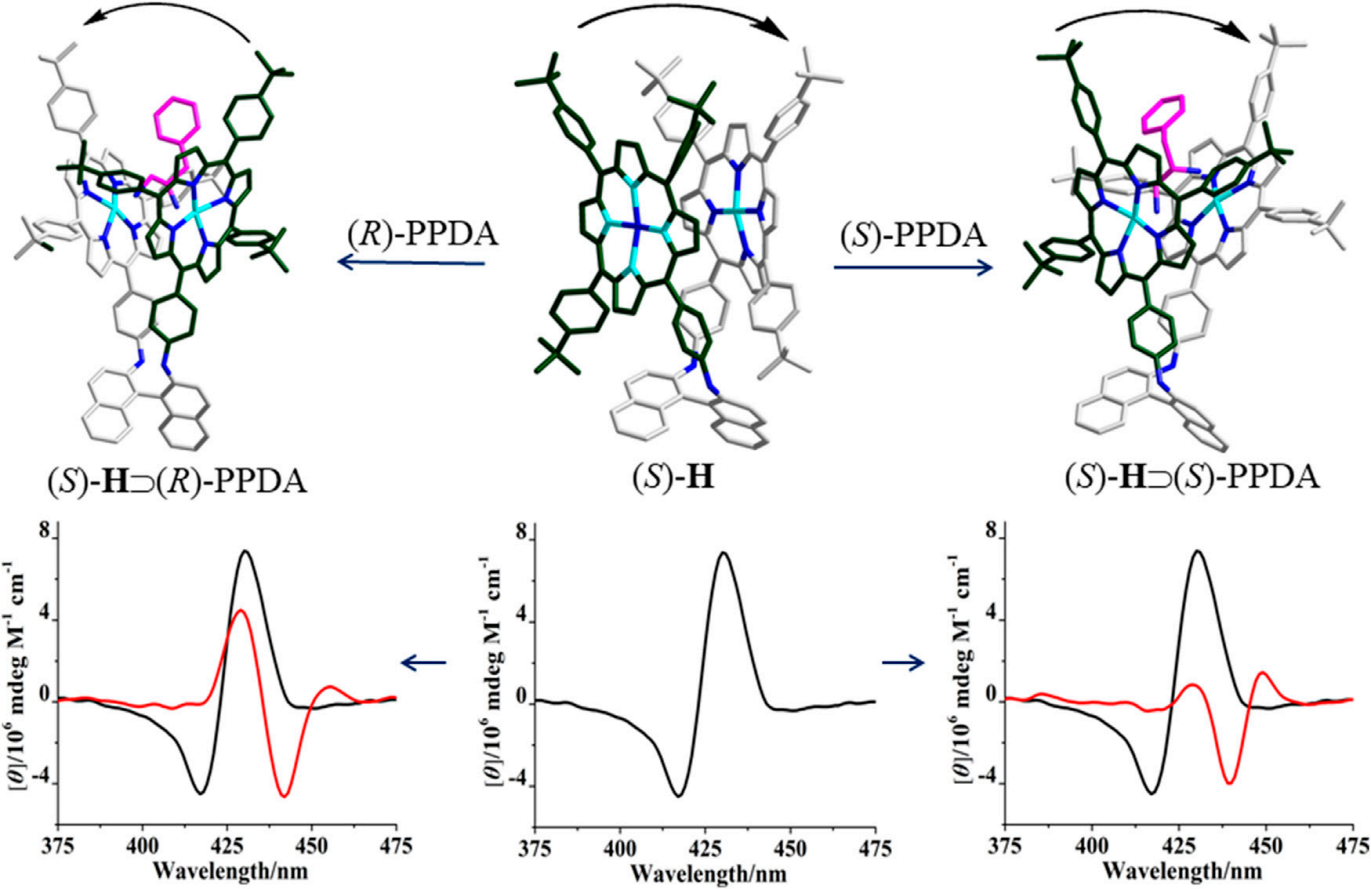


## Introduction

Chiral phenomenon widely exists in nature such as proteins, polysaccharides and nucleic acids, playing an important role in the development and evolution of life. Modulating the chirality of supramolecular system is pivotal owing to its significance in chemistry (de Jong et al., 2004; Liu et al., 2014; Liu et al., 2015) and material science (Jung et al., 2013; Dou et al., 2020; Wang and Feng, 2018). Supramolecular chirality modulation is crucial for understanding the stereostructure and function of chiral compounds (van Dijken et al., 2014; Liu et al., 2016). In addition, supramolecular chirality modulation has important applications in molecular recognition (Wang et al., 2017; Wu et al., 2019), asymmetric catalysis (Dydio et al., 2011; Hong et al., 2016), chiroptical devices (Wang et al., 2019) and medicines (Weatherly et al., 2017; Li et al., 2019; Ma et al., 2019).

1,1ʹ-binaphthyl derivatives are one of the most important class of C2-symmetric compound. The pure enantiomers of 1,1ʹ-binaphthyl derivatives have inherent chiral induction abilities due to their rigid *C*_2_ symmetrical structures. In addition, the structures of 1,1ʹ-binaphthyl derivatives are highly tunable since the 2-, 3-, 4-, 6-, 7-, 8- and 9-positions can be systematically modified by introducing functional groups. As a result, they have found extensive applications in the development of optoelectric materials (Yu et al., 2011), optical sensors for molecular recognition (Yu et al., 2013) and asymmetric catalysis (Chen et al., 2003).

On the other hand, porphyrins are highly involved in supramolecular chirality systems owing to the unique photonic and electronic activities (Peng et al., 2008; Stefanelli et al., 2019; Mondal and Rath, 2020). In particular, covalently linked bisporphyrin hosts can bind with guest molecules *via* non-covalent interactions, and their CD spectra are sensitive to the corresponding allosteric effects (Hu et al., 2018; Dhamija et al., 2020). As a result, achiral bisporphyrins can be applied to the exciton coupled circular dichroism (ECCD) protocol for determining the absolute configurations of chiral guest molecules (Hayashi et al., 2015; Hu et al., 2017).

Furthermore, by connecting two porphyrin monomers with chiral spacers, chiral bisporphyrins can be obtained, which are of significance for the development of chiral discrimination systems. In this respect, we reported 1,1′-bi-2-naphthol (BINOL) linked porphyrin dimers for the study of intermolecular chirality modulation and chiral discrimination toward a range of model diamines (Lu et al., 2017a). The results indicated that the enantioselectivity and chiral sensing properties of chiral bisporphyrin hosts can be gradually tuned by varying the connecting units between porphyrin and BINOL moieties.

**SCHEME 1:**
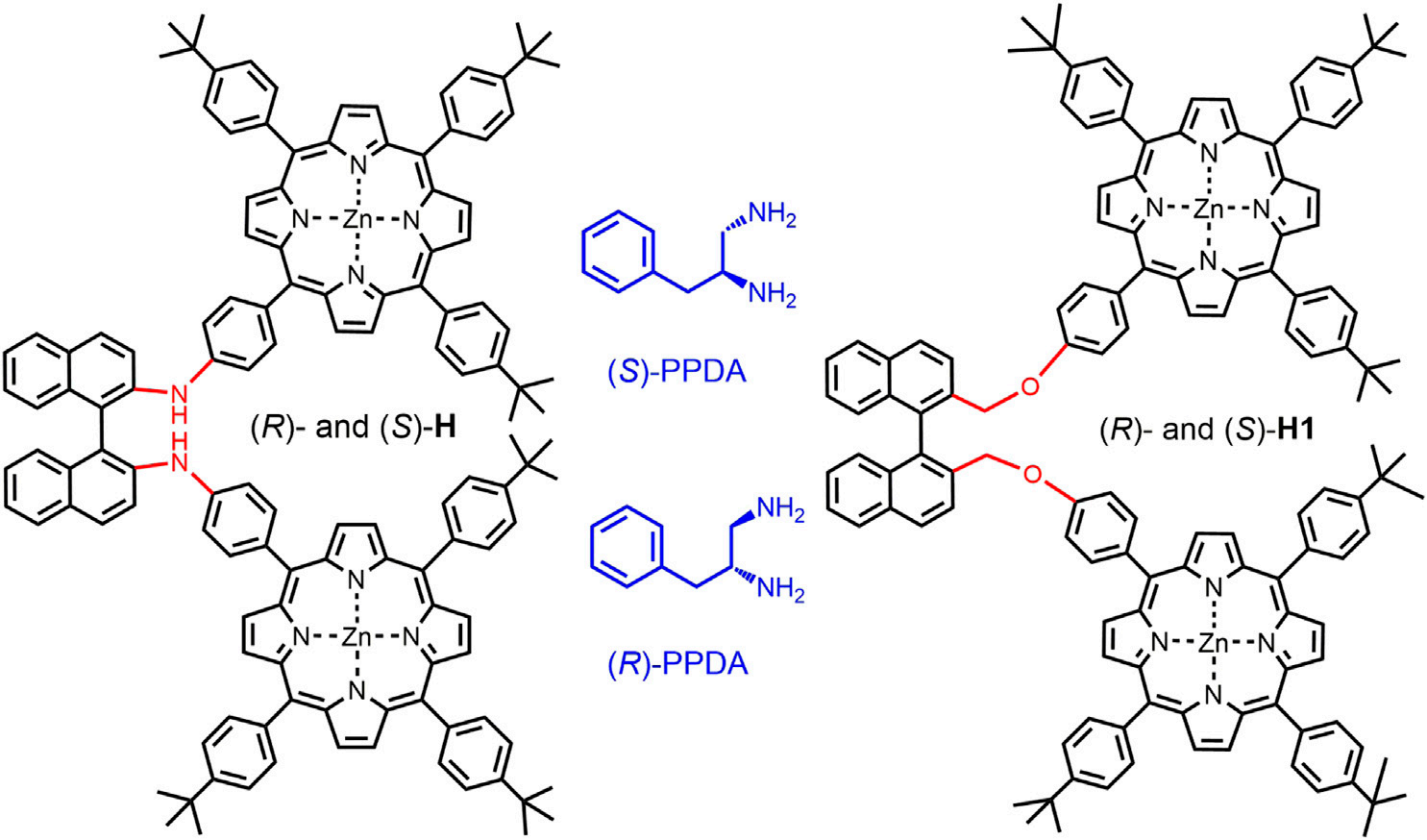
Structures of (*R*)-/(*S*)-**H**, (*R*)-/(*S*)-**H1** and (*R*)-/(*S*)-PPDA.

Herein, we present new dimeric porphyrin hosts (*R*)-/(*S*)-**H**, which were formed by linking two porphyrin units via a (*R*)- or (*S*)-2,2ʹ-diamino-1,1ʹ-binaphthyl (Scheme 1). The supramolecular complexation and intermolecular chirality modulation with (*R*)-/(*S*)-PPDA were studied by electronic absorption, fluorescence, ^1^H NMR and CD spectroscopies with the help of DFT calculations. The importance of the linking units in bisporphyrin hosts for chiral recognition was revealed by comparing with the previously reported hosts (*R*)-/(*S*)-**H1** (Lu et al., 2017b).

## Results and Discussion

### Synthesis and Characterization

The new chiral bisporphyrins (*R*)-/(*S*)-**H** were synthesized by Buchwald-Hartwig reaction of (*R*)- or (*S*)-2,2ʹ-diamino-1,1ʹ-binaphthyl with the mono-brominated Zn(II) porphyrinate. The target compounds were obtained with moderate yields (34–35%) and adequately characterized by Mass, NMR and UV-Vis absorption spectral data (see the experimental section and the Supporting Information for details, Supplementary Scheme S1 and Supplementary Figures S1,S2).

### UV-Vis Spectrophotometric and Fluorescence Titration

**FIGURE 1:**
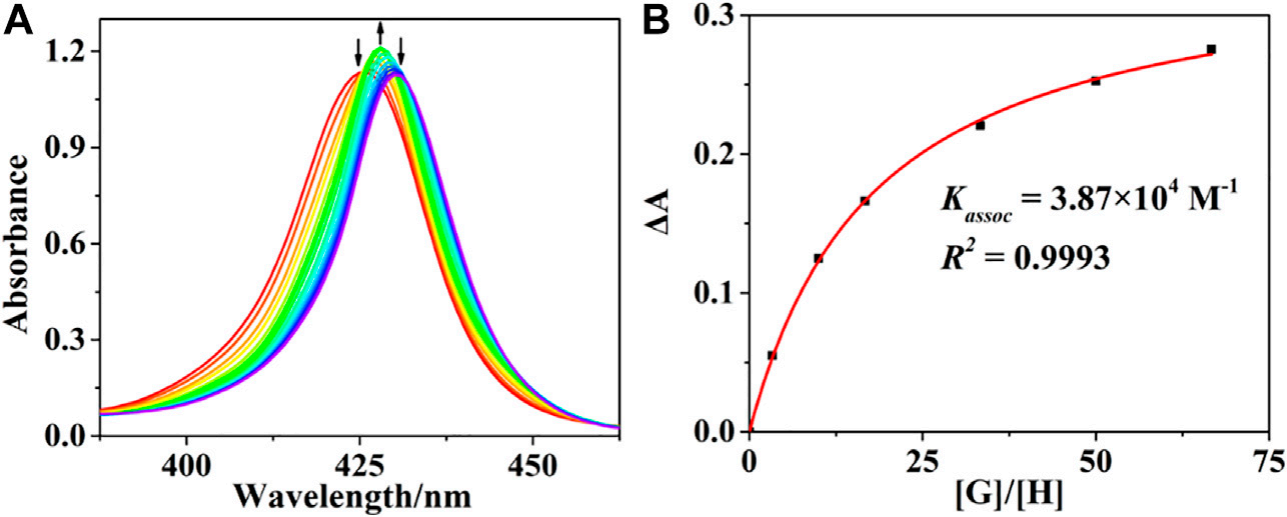
**(A)** UV-Vis titration profiles of (*R*)-**H** with (*S*)-PPDA. [(*R*)-**H**] = 1.5 × 10^–6^ M; [(*S*)-PPDA]/[(*R*)-**H**] = 0–670. **(B)** Changes in Δ*A* at 420 nm for evaluating *K*_assoc_, the solid line represents the non-liner least square fit for 1:1 complexation.

The interactions of (*R*)-/(*S*)-PPDA with host (*R*)-/(*S*)-**H** were first monitored by UV-Vis spectrophotometric titration at 298 K in CHCl_3_ (Figure 1 and Supplementary Figure S3). Upon adding (*S*)-PPDA to (*R*)-**H** gradually, the B absorption band increased and the absorption maxima redshifted from 425 to 428 nm at the lower guest concentration range (0–67 equiv). An isosbestic point appears at 426 nm, suggesting the domination of a host-guest complex. Considering the ditopic feature of (*S*)-PPDA and (*R*)-**H**, a 1:1 sandwich host-guest complex ((*R*)-H⊃(*S*)-PPDA) can be put forward, where a PPDA binds to the two porphyrin moieties by Zn-N coordination (Lu et al., 2017a). The sandwich complexes were found stable up to 67 equiv of (*R*)-/(*S*)-PPDA were added, and the association constants (*K*_*assoc*_) of (*R*)-H⊃(*S*)-PPDA and (*R*)-H⊃(*R*)-PPDA were evaluated as 3.87 × 10^4^ M^−1^ and 2.88 × 10^4^ M^−1^, respectively (Thordarson, 2011) (Figure 1B and Supplementary Figure S4). These association constant values are significantly lower than those of the previously reported host **H1** with PPDA (Lu et al., 2017a), which is obviously due to the decreased length and flexibility of the linking units in the present bisporphyrin host **H**. Therefore, further addition of (*R*)-PPDA or (*S*)-PPDA (67–670 equiv) to the host (*R*)-**H** may trigger the conversion to 1:2 open complexes (*R*)-**H**@[(*R*)-PPDA]_2_ and (*R*)-**H**@[(*S*)-PPDA]_2_ respectively, which can be supported by the further 2 nm redshift of the Soret band and the decrease in its intensity.

**FIGURE 2:**
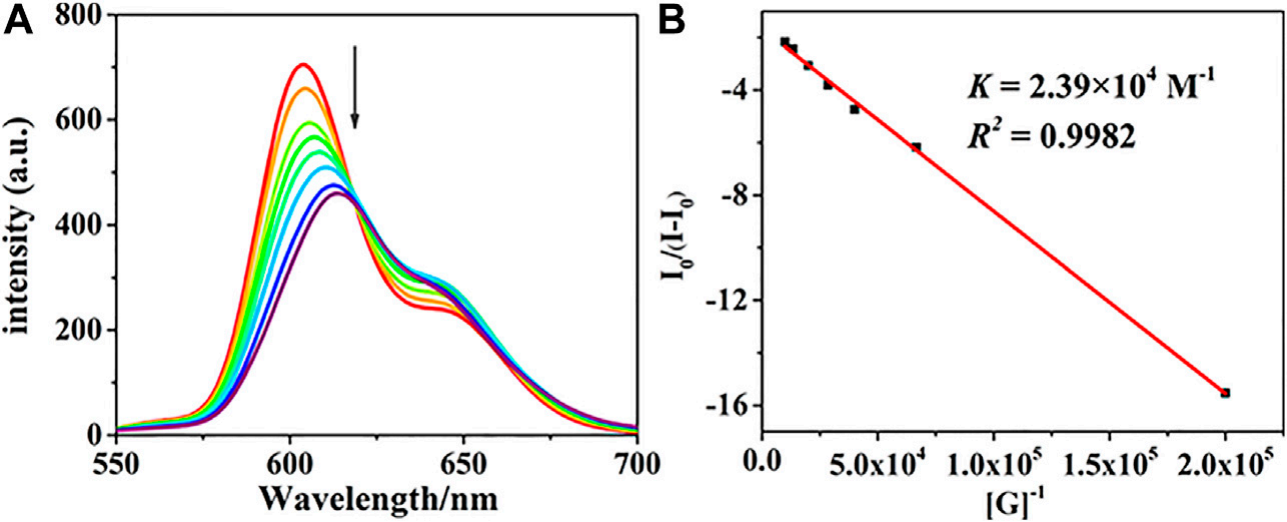
**(A)** The fluorescence titration profiles of (*R*)-**H** with (*R*)-PPDA [(*R*)-**H**] = 1.5 × 10^–6^ M; [(*R*)-PPDA]/[(*R*)-**H**] = 0–67, *λ*_*Ex*_ = 415 nm. **(B)** The Benesi-Hildebrand plot monitored at 604 nm.

The fluorescence response behaviors of the chiral porphyrin dimer (*R*)-**H** toward (*R*)- and (*S*)-PPDA were also recorded at 298 K in CHCl_3_ with the excitation at 415 nm. When a solution of (*R*)-**H** (1.5 × 10^–6^ M) was treated with (*R*)-PPDA (0–67 equiv), the fluorescence intensity greatly decreased with a redshift from 604 nm to 617 nm (Figure 2A). The Benesi-Hildebrand plot (Figure 2B) shows a linear relationship in the [G]/[H] range of 0–67 equiv, also suggesting a 1:1 stoichiometry for the stable supramolecular complex (*R*)-**H**⊃(*R*)-PPDA. The *K*_*assoc*_ was estimated to be 2.39 × 10^4^ M^−1^ for (*R*)-**H**⊃(*R*)-PPDA (Hariharan et al., 2007; Jiao et al., 2018). The titration profiles of (*R*)-**H** with (*S*)-PPDA resemble those of (*R*)-**H** with (*R*)-PPDA, and the *K*_*assoc*_ for (*R*)-**H**⊃(*S*)-PPDA was obtained as 4.09 × 10^4^ M^−1^ (Supplementary Figure S5,S6). The *K*_*assoc*_ values are in good agreement with those obtained from the UV-Vis spectrophotometric titration.

### ^1^H NMR Titration

**FIGURE 3:**
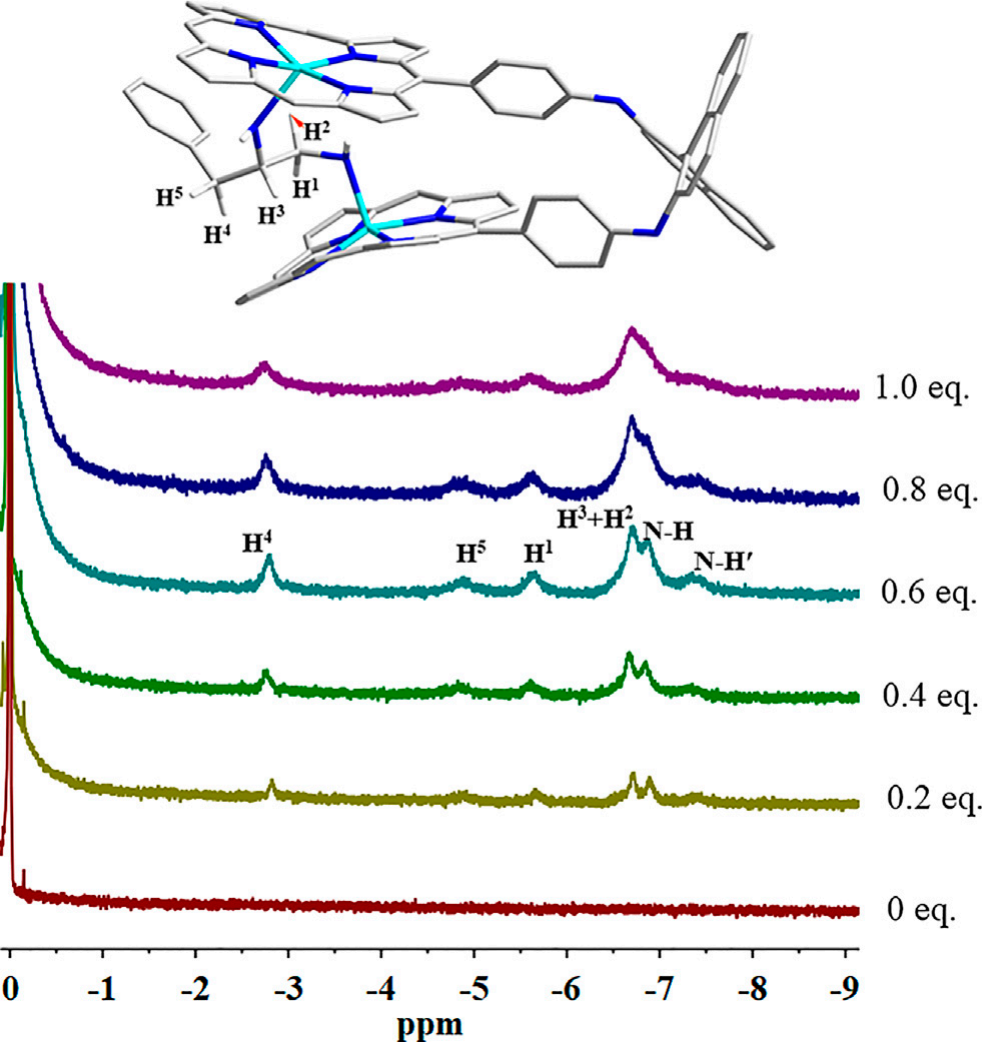
^1^H NMR spectra of (*S*)-**H** (0.75 mM) in the presence of (*S*)-PPDA.

The binding of (*S*)-PPDA to the chiral bisporphyrin hosts was also probed by ^1^H NMR spectroscopy at 298 K in CDCl_3_ (Figure 3 and Supplementary Figure S7). Upon the addition of 0.4 equiv of (*S*)-PPDA to (*S*)-**H**, the signals of the free host completely disappeared, suggesting the fast exchange between free and bound (*S*)-**H** molecules. Meanwhile, the signals of (*S*)-PPDA moved to high field (from δ 2.97–1.25 to δ −2.78 to −7.38 ppm). The complexation induced shift (CIS) values of the bound guest (*S*)-PPDA (Δ*δ* = *δ*_bonded PPDA_ −*δ*_free PPDA_) are in the range of −5.33 to −9.56 (Table 1), which are reasonably close to those of a diamine tweezered by a dimeric Zn(II) porphyrin (Pintre et al., 2012; Lu et al., 2017a). The observations further support the formation of the 1:1 sandwich complex (*S*)-**H**⊃(*S*)-PPDA, where the diamine molecule is shielded by the two porphyrin rings. Compared with the ^1^H NMR titration results of (*S*)-**H1** with (*S*)-PPDA (Lu et al., 2017a), the proton signals of PPDA in (*S*)-**H**⊃(*S*)-PPDA were broad and weak due to the low binding affinities of (*S*)-PPDA with (*S*)-**H**. Furthermore, the CIS values (|Δ*δ*|) of almost all the (*S*)-PPDA protons were much larger than those in (*S*)-**H1**⊃(*S*)-PPDA, which indicated that the PPDA guest was shielded by stronger ring-current effect from the two porphyrin rings in (*S*)-**H**. These observations can be attributed to the different length and flexibility of the linking units between binaphthalene and porphyrin for (*S*)-**H1** and (*S*)-**H**.

**TABLE 1:** ^1^H NMR signals of (*S*)-PPDA and the complexation induced shift values (Δ*δ* = *δ*_bonded PPDA_ − *δ*_free PPDA_), at 298 K in CDCl_3_.

Proton	Free (*S*)-PPDA *δ*/ppm	(*S*)-H⊃(*S*)-PPDA *δ*/ppm	Δ*δ*/ppm
N-H	1.25	−6.89	−8.14
N-H′	1.25	−7.38	−8.63
H^1^	2.82	−5.63	−8.45
H^2^	2.78	−6.68	−9.46
H^3^	2.97	−6.68	−9.65
H^4^	2.55	−2.78	−5.33
H^5^	2.49	−4.87	−7.36

### Circular Dichroism Response

**FIGURE 4:**
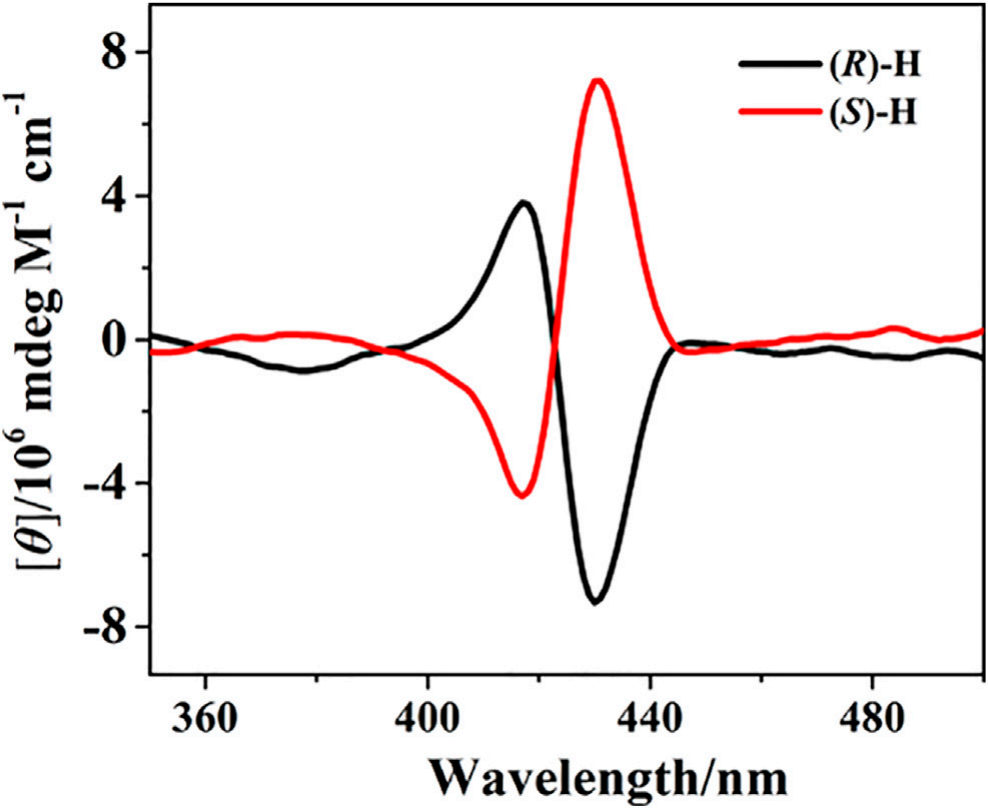
CD spectra of bisporphyrin hosts (*R*)-/(*S*)-**H** in CHCl_3_ at 298 K.

The electronic circular dichroism (CD) spectra of (*R*)-**H** and (*S*)-**H** were recorded in chloroform at 298 K. In the porphyrin Soret band region, split Cotton effects are observed at 430 and 418 nm with opposite signs for (*R*)- and (*S*)-**H**. (*R*)-**H** shows negative CD couplets, while (*S*)-**H** exhibits positive CD couplets, forming perfect mirror images in the CD spectra (Figure 4). The sign of CD couplets can be directly correlated to the chirality of the linkage by the exciton coupled circular dichroism (ECCD) theory (Pescitelli et al., 2014).

**FIGURE 5:**
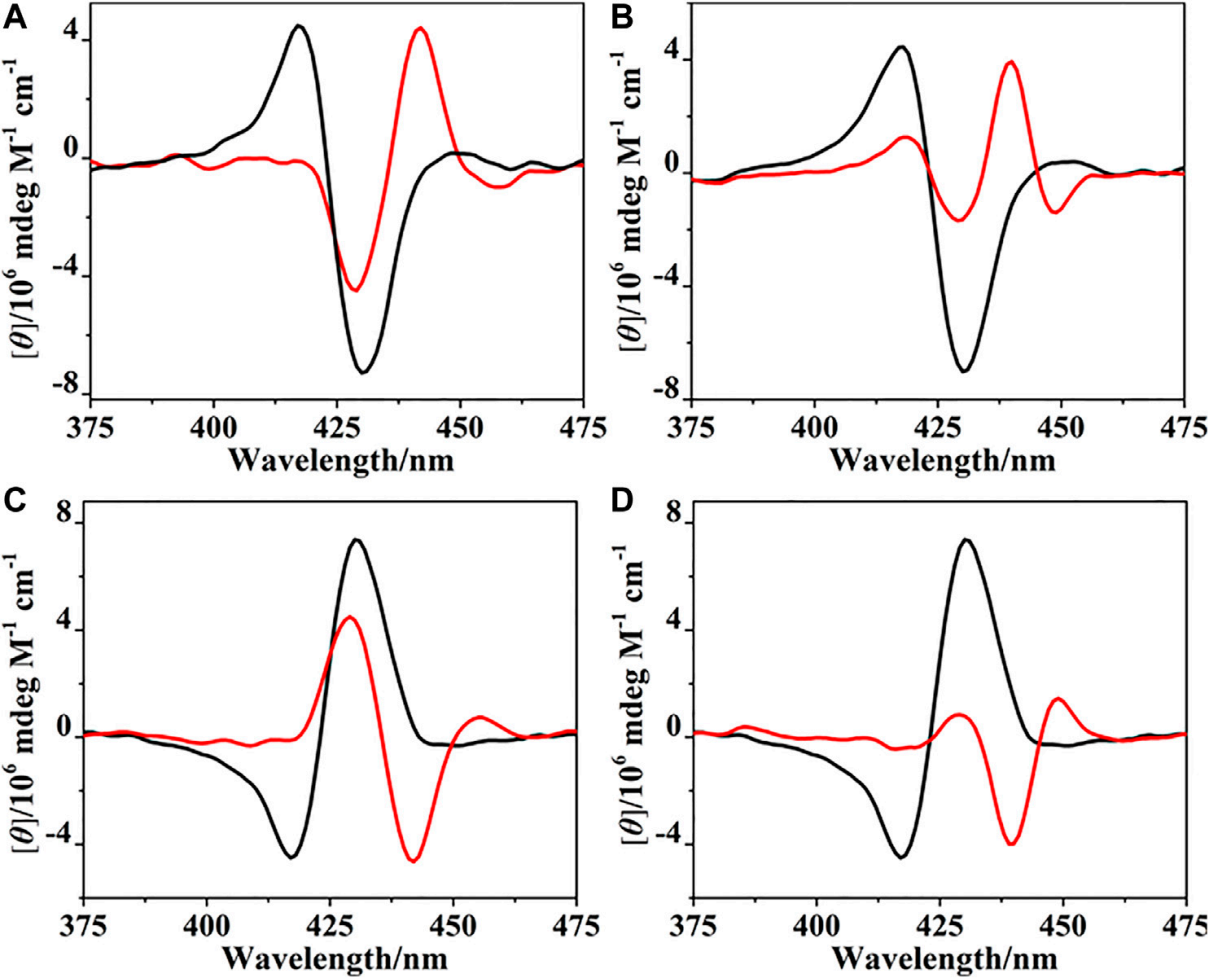
CD spectra of (*R*)-**H** in the absence (black) and presence (red) of PPDA (67 equiv): **(A)** (*S*)-PPDA, **(B)** (*R*)-PPDA; CD spectra of (*S*)-**H** in the absence (black) and presence (red) of PPDA (67 equiv): **(C)** (*R*)-PPDA, and **(D)** (*S*)-PPDA).

The binding of (*R*)- and (*S*)-PPDA to the bisporphyrin hosts (*R*)-/(*S*)-**H** was further investigated by CD spectroscopy. Upon addition of (*S*)-PPDA (0–67 equiv) to (*R*)-**H**, the CD signals at 430 and 418 nm decreased, new signals appeared at 442 and 428 nm (Figure 5A). Notably, the negative Cotton effect of (*R*)-**H** is transformed to a positive Cotton effect with about 10 nm red shift and decreased amplitude. The remarkable CD inversion can be assigned to the binding-induced allosteric effects along with the formation of 1:1 complex (*R*)-**H**⊃(*S*)-PPDA. In comparison, the titration of (*R*)-**H** with (*R*)-PPDA induced only a red shift and decrease of the negative CD couplets, while no CD inversion was observed along with the formation of (*R*)-**H**⊃(*R*)-PPDA (Figure 5B). These changes were slightly different with the CD response of (*R*)-**H1** in the presence of (*S*)-/(*R*)-PPDA (Lu et al., 2017a). For (*R*)-**H1,** when titrated with (*S*)-PPDA the CD signals transformed to a *positive Cotton effect* with a red shift (13 nm) and increased amplitude, when titrated with (*R*)-PPDA the CD signals also showed a similar red shift and a remarkably decreased amplitude. The titration results of (*S*)-**H** with (*R*)-/(*S*)-PPDA are similar to that of (*R*)-**H**. The obtained CD spectra of (*S*)-**H**⊃(*R*)-PPDA and (*S*)-**H**⊃(*S*)-PPDA are the mirror images of those of (*R*)-**H**⊃(*S*)-PPDA and (*R*)-**H**⊃(*R*)-PPDA, respectively (Figure 5).

According to the CD spectral changes induced by the supramolecular binding for (*R*)-/(*S*)-**H** and (*R*)-/(*S*)-**H1** (Lu et al., 2017a), it is obvious that (*S*)-PPDA induces *positive CD couplets*, while (*R*)-PPDA leads to *negative CD couplets*, thus the final CD signs of the 1:1 host-guest complexes are dominated by the stereostructure of diamines. When the bisporphyrin binds a diamine with different chirality, CD inversion will be observed. Nevertheless, for (*R*)-/(*S*)-**H1**, we noted that the formation of (*R*)-**H1**⊃(*R*)-PPDA and (*S*)-**H1**⊃(*S*)-PPDA resulted in extremely small CD amplitudes, which could make it difficult for determining the CD signs (Lu et al., 2017a). While for (*R*)-/(*S*)-**H**, after the formation of (*R*)-**H**⊃(*R*)-PPDA and (*S*)-**H**⊃(*S*)-PPDA, the CD signals still have moderate intensity and the CD signs can be easily determined, which is favorable for chiral optical sensing.

### DFT Molecular Modeling

**FIGURE 6:**
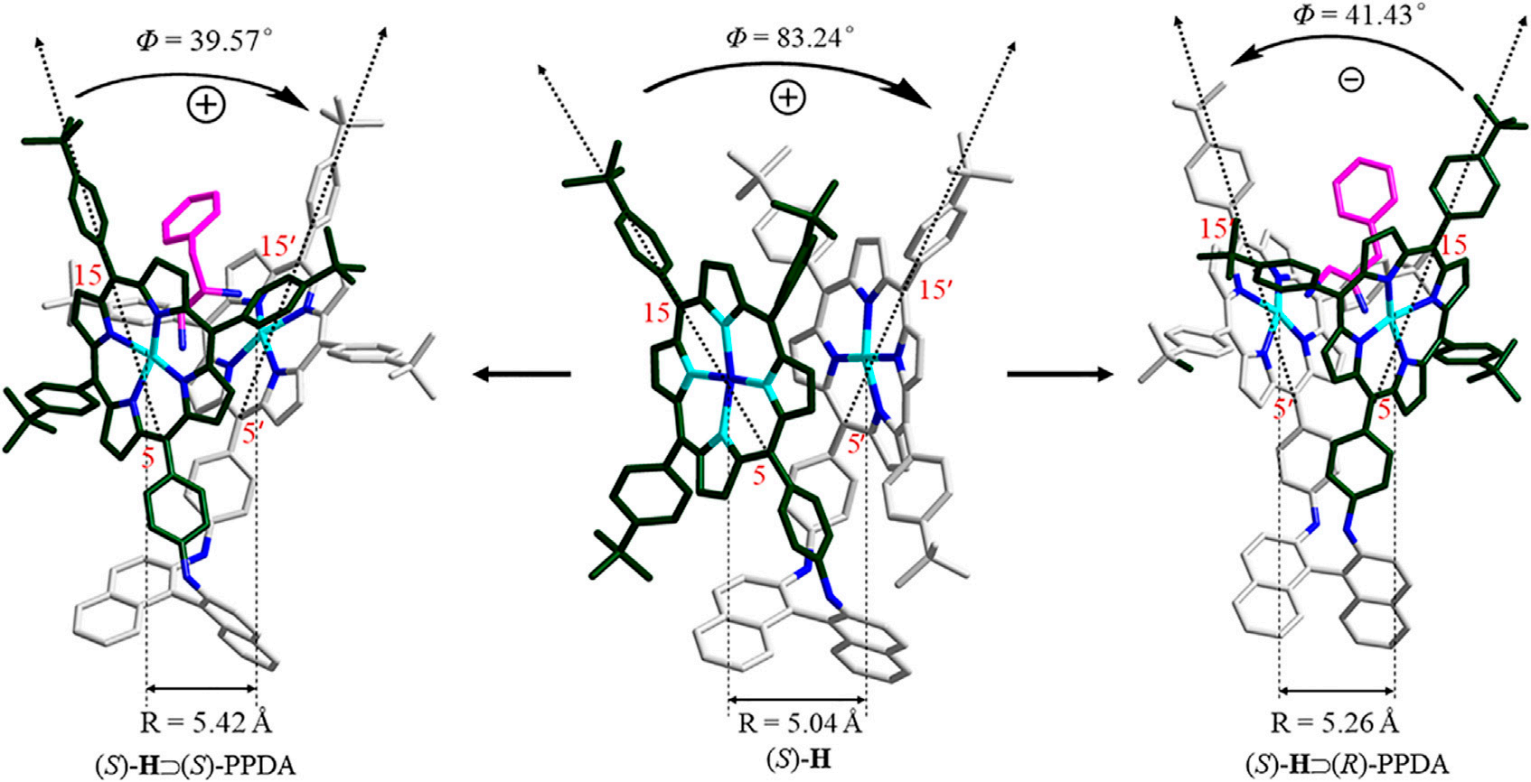
DFT optimized structures of (*S*)-**H**, (*S*)-**H**⊃(*S*)-PPDA and (*S*)-**H**⊃(*R*)-PPDA.

To further rationalize the CD spectral change, DFT molecular modeling was performed at the B97D/6-31G(D) level. As shown in the optimized molecular structures (Figure 6 and Table 2), the free host (*S*)-**H** exhibits a clockwise chiral twist (+83.24°) between the coupled effective electric transition moments (EETMs) across the C5/C15 of porphyrin rings, which is in line with the positive CD couplets of (*S*)-**H** (Figure 4). The formation of stable (*S*)-**H**⊃(*S*)-PPDA and (*S*)-**H**⊃(*R*)-PPDA induces significant changes in the coupled EETMs. For (*S*)-**H**⊃(*S*)-PPDA, the twist angle between the two coupled EETMs decreases from +83.24° to +39.57°, though the clockwise direction is unchanged. In contrast, the twist angle between the coupled EETMs for (*S*)-**H**⊃(*R*)-PPDA inverted to anticlockwise (−41.43°), which is consistent with the observed CD inversion phenomenon for this system. Moreover, the relative distance between the two interacting porphyrin moieties (i.e. the Zn-Zn distance) is also altered by the formation of 1:1 complexes. The Zn-Zn distance is increased from 5.04 Å for (*S*)-**H** to 5.42 and 5.26 Å for (*S*)-**H**⊃(*S*)-PPDA and (*S*)-**H**⊃(*R*)-PPDA, respectively. The CD amplitude is not only inversely proportional to the square of interporphyrin distance, but also is a function of the twist angle between the two coupled EETMs (Pescitelli et al., 2014). The maximum CD amplitude appears at a twist angle of 70° (Nina et al., 2012). The free host (*S*)-**H** possesses a larger Zn-Zn distance (5.04 Å) and a twist angle (+83.24°) than the free host (*S*)-**H1** (3.48 Å and +21.22°), probably due to the relatively short linkage and rigid structure. After the formation of supramolecular complexes with (*R*)-/(*S*)-PPDA, the Zn-Zn distances of (*S*)-**H**⊃(*S*)-PPDA and (*S*)-**H**⊃(*R*)-PPDA get slightly increased while the twist angles get decreased, both leading to the decrease of the corresponding CD amplitude. Thus, the observed decrease in CD intensity for the supramolecular complexes (Figure 5) is in accordance with the theoretical prediction.

**TABLE 2:** Parameters of the optimized molecular structures of (*S*)-**H**, (*S*)-**H**⊃(*S*)-PPDA, (*S*)-**H**⊃(*R*)-PPDA, (*S*)-**H1**, (*S*)-**H1**⊃(*S*)-PPDA and (*S*)-**H1**⊃(*R*)-PPDA.

Parameter	R (Å)	*Φ* (°)
(*S*)-**H**	5.04	+83.24
(*S*)-**H**⊃(*S*)-PPDA	5.42	+39.57
(*S*)-**H**⊃(*R*)-PPDA	5.26	−41.43
(*S*)-**H1** ^a^	3.48	+21.22
(*S*)-**H1**⊃(*S*)-PPDA^a^	6.10	+50.52
(*S*)-**H1**⊃(*R*)-PPDA^a^	5.18	−31.73

^a^ Adopted from (Lu et al., 2017a).

However, this is not the case for (*S*)-**H1** (Lu et al., 2017a). Upon binding to (*R*)-/(*S*)-PPDA, the Zn-Zn distances increased significantly from 3.48 ((*S*)-**H1**) to 6.10 ((*S*)-**H1**⊃(*S*)-PPDA) and 5.18 Å ((*S*)-**H1**⊃(*R*)-PPDA) respectively, which can induce a decrease of the CD amplitude. Meanwhile the twist angles also increased from +21.22° to +50.52° ((*S*)-**H1**⊃(*S*)-PPDA) and −31.73° ((*S*)-**H1**⊃(*R*)-PPDA) respectively, which may tend to an increase in the CD amplitude. As a result, the prediction of CD amplitude change for (*S*)-**H1** becomes complicated and uncertain due to the two opposite factors.

## Conclusion

In summary, we have presented porphyrin dimers (*R*)-/(*S*)-**H** and investigated their complexation abilities with (*R*)-/(*S*)-PPDA by using UV-Vis absorption, fluorescence and NMR titrations. At low guest concentration (0–67 equiv), 1:1 sandwich host-guest complexes were formed. The intermolecular chirality modulation process has been monitored by CD spectroscopy. The binding process afforded obvious CD spectral change, and the CD signs of 1:1 sandwich host-guest complexes are dominated by the stereostructure of guest molecules. The sensitive CD responses can be attributed to the short linking units and the binding-induced allosteric effects according to the DFT molecular modeling. The present results indicate that the chiral bisporphyrin hosts have great potential as chiral optical probes.

## Data Availability

The original contributions presented in the study are included in the article/Supplementary Material, further inquiries can be directed to the corresponding authors.
