# Supplementary material for: Intermolecular Chirality Modulation of Binaphthalene-Bridged Bisporphyrins With Chiral Diamines
Source: Front Chem. 2021 Feb 12;8:611257. doi: 10.3389/fchem.2020.611257 (PMC7919525; doi:10.3389/fchem.2020.611257)
Supplement: Supplementary file 1 [file datasheet1.docx]

**Supporting Information**

**Intermolecular Chirality Modulation of Binaphthalene-Bridged Bisporphyrins with Chiral Diamines**

**Wenxin Lu,^a,b^ Lei Gong,^b^ Chaorui Su,^b^ Qibao Wang,^a^ Qing Ling,^a^ Peng Wang,^a^ Dongdong Qi^b^ and Yongzhong Bian^b^**

*^a^ College of Chemical and Biological Engineering, Shandong University of Science and Technology, Qingdao, 266590, China*

*^b^Beijing Key Laboratory for Science and Application of Functional Molecular and Crystalline Materials, Department of Chemistry, University of Science and Technology Beijing, Beijing 100083, China*

**Content**

1. Chemicals and instruments, page S3.
2. Synthesis and characterization, pages S4-S7.
3. UV-Vis spectrophotometric titration, page S8 and S9.
4. Fluorescence titration, page S10 and S11.
5. ^1^H NMR titration, page S12.
6. Computational details, page S13.
7. References, page S14.

1. Chemicals and Instruments

Column chromatography was carried out on silica gel (200-300 mesh, Qingdao Ocean Chemicals) with the indicated eluent. Toluene and N,N-dimethylformamide (DMF) were freshly distilled from CaH_2_ under nitrogen. 5-(4-bromophenyl)-10,15,20-tris(4-*tert*-butylphenyl)porphyrin (Zn(TTBPP)Br) was prepared according to the published procedures^[S1]^. All other reagents and solvents were used as received.

^1^H NMR spectra were recorded on a Bruker DPX 400 spectrometer (400 MHz) in CDCl_3_ and the chemical shifts were reported relative to internal SiMe_4_. MALDI-TOF mass spectra were taken on a Bruker Microflex^TM^ LRF spectrometer with dithranol as the matrix. Elemental analyses were performed on a Perkin-Elmer Model 2400 analyzer. Electronic absorption spectra were recorded on a Lambda 750 spectrophotometer. Steady-state fluorescence spectroscopic studies were performed on a Hitachi F4500 fluorophotometer. CD spectra were recorded on a JASCO J-1500 spectropolarimeter.

2. Synthesis and Characterization

**Scheme S1**. Synthesis of the zinc(II) bisporphyrin hosts **H**.

1. Synthesis of (*S*)-**H**

Cs_2_CO_3_ (97 mg, 0.30 mmol), Xantphos (23.12 mg, 0.04 mmol), Pd_2_(dba)_3_ (9.15 mg, 0.01 mmol) were added to a stirred solution of **P1** (231 mg, 0.25mmol) in dry toluene (15 ml). Then a solution of (*S*)-(-)-2,2ʹ-diamino-1,1ʹ-binaphthyl (28.4 mg, 0.10 mmol) in dry toluene (10 mL) was added dropwise. The mixture was heated to 100 °C and reacted under nitrogen for 24 h. After evaporation of the solvent, the residue was chromatographed on a silica gel column using CHCl_3_/n-hexane (3:1, in v/v) as the eluent. The second fraction containing the target compound was collected and evaporated. Repeated chromatography followed by recrystallization from chloroform and methanol gave pure (*S*)-**H** as a purple powder (69 mg, 35%). ^1^H NMR (CDCl_3_, 400 MHz, 293 K): *δ* 8.85 (d, 8H, *J* = 4.4 Hz), 8.78 (d, 4H, *J* = 4.4 Hz), 8.64 (d, 4H, *J* = 4.4 Hz), 8.20 (d, 2H, *J* = 9.2 Hz), 8.12(m, 10H), 7.99 (d, 2H, *J* = 8.0 Hz), 7.93 (d, 4H, *J* = 8.0 Hz), 7.69 (d, 4H, *J* = 7.6 Hz), 7.64 (t, 4H, *J* = 8.8 Hz), 7.51 (d, 4H, *J* = 7.6 Hz), 7.44 (t, 10H, *J* = 11.4 Hz), 7.21 (d, 4H, *J* = 9.2 Hz), 6.17 (s, 2H), 1.99 (s, 6H), 1.59 (s, 18H), 1.43 (s, 30H); UV-*vis* (CHCl_3_): λ_max_ (log*ε*) 426 (5.83), 552 (4.64), 594 nm (4.19). MALDI-TOF-MS m/z calcd. for C_132_H_116_N_10_Zn_2_: (M^+^) 1973.21; Found: 1973.11. Anal. calcd (%) for C_132_H_116_N_10_Zn_2_ : C, 80.35; H, 5.93; N, 7.10. Found: C, 80.42; H, 5.88; N, 7.06.

1. Synthesis of (*R*)-**H**

(*R*)-**H1** was synthesized following the above method for (*S*)-**H1**, starting from (*R*)-(+)-2,2ʹ-diamino-1,1ʹ-binaphthyl (28.4 mg, 0.10 mmol) instead of (*S*)-(-)-2,2ʹ-diamino-1,1ʹ-binaphthyl. (*R*)-**H** was obtained as a purple powder (67 mg, 34%).


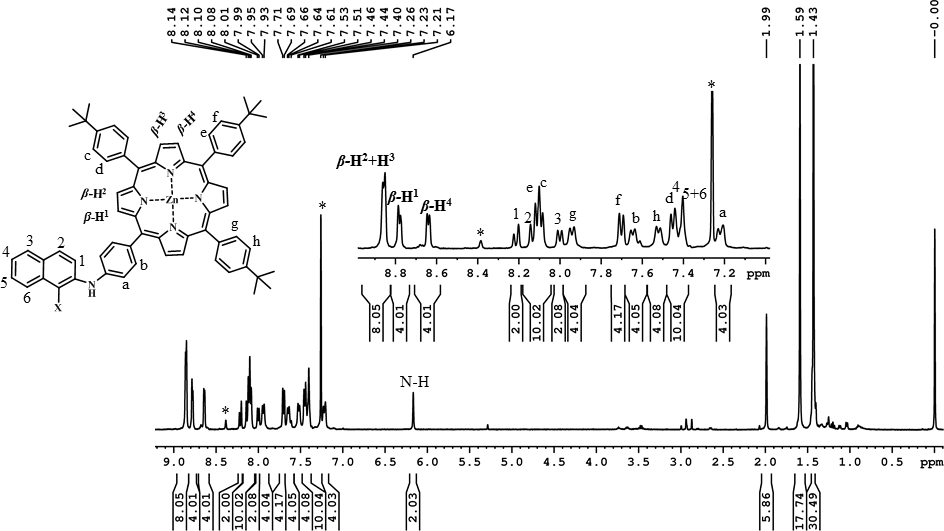


**Figure S1**. ^1^H NMR spectrum of **H** in CDCl_3_ at 293 K. * indicate the residual solvent signals.


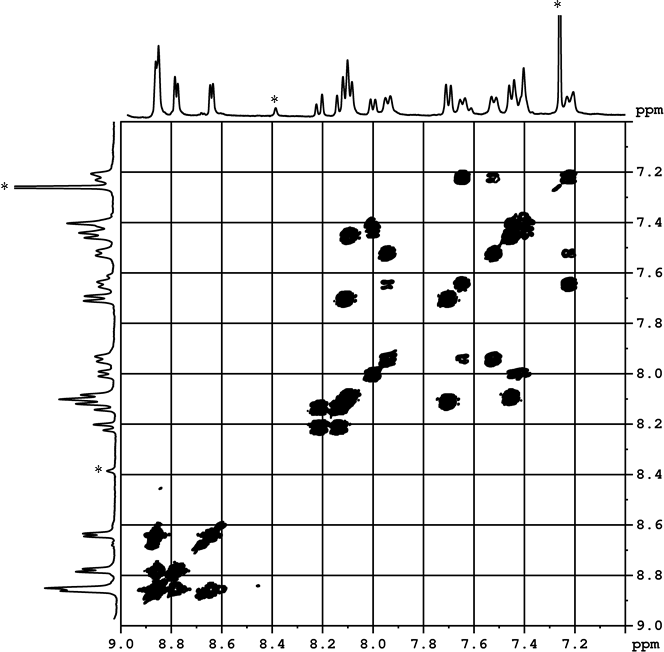


**Figure S2**. ^1^H-^1^H COSY spectra of **H** in CDCl_3_ at 293 K. * indicate the residual solvent signals.

**UV-Vis Spectrophotometric Titration.**

Method for Evaluation of Association Constants (*K_assoc_*)

The association constant *K_assoc_* for the 1:1 complexes was derived by using the non-linear curve fitting based on the equation:

*ΔAbs* = (A_∞_(1+*K_assoc_*[G]+*K_assoc_*[H]) - (A_∞_^2^(*K_assoc_*[G]+*K_assoc_*[H]+1)^2^-4*K_assoc_*^2^[H]*[G] A_∞_^2^)^0.5^)/2*K_assoc_*[H]

Where [G] and [H] represent [Guest]_total_ and [Host]_total_, respectively; A_∞_ denotes *ΔAbs* at 100% complexation; A_∞_ and *K_assoc_* are parameters. ^[S2]^


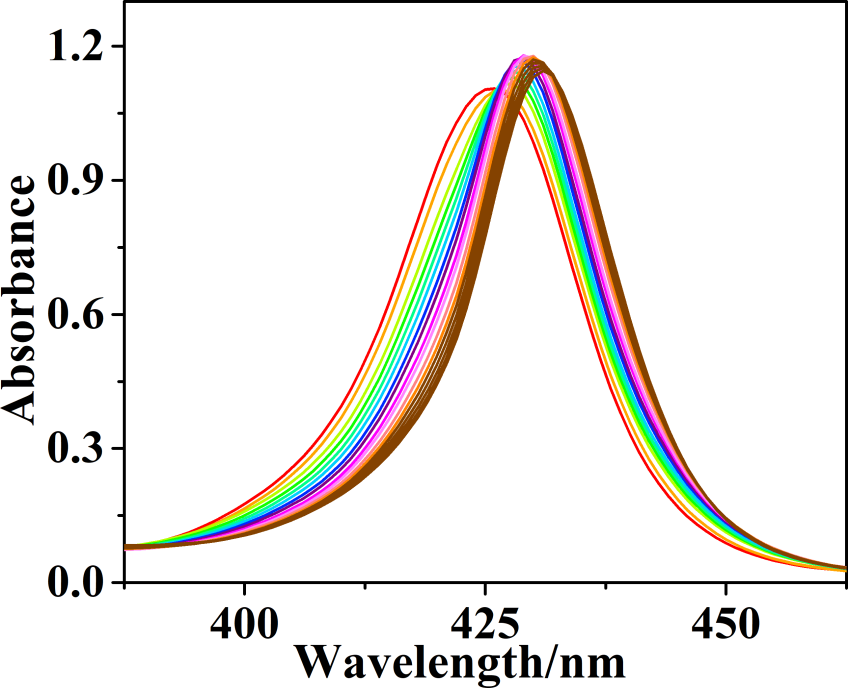


**Figure S3.** Spectral change upon titration of (*R*)-**H** with (*R*)-PPDA in CHCl_3_ at 298 K. [(*R*)-**H**] = 1.5 × 10^−6^ M; [(*R*)-PPDA]/[(*R*)-**H**] = 0−670.


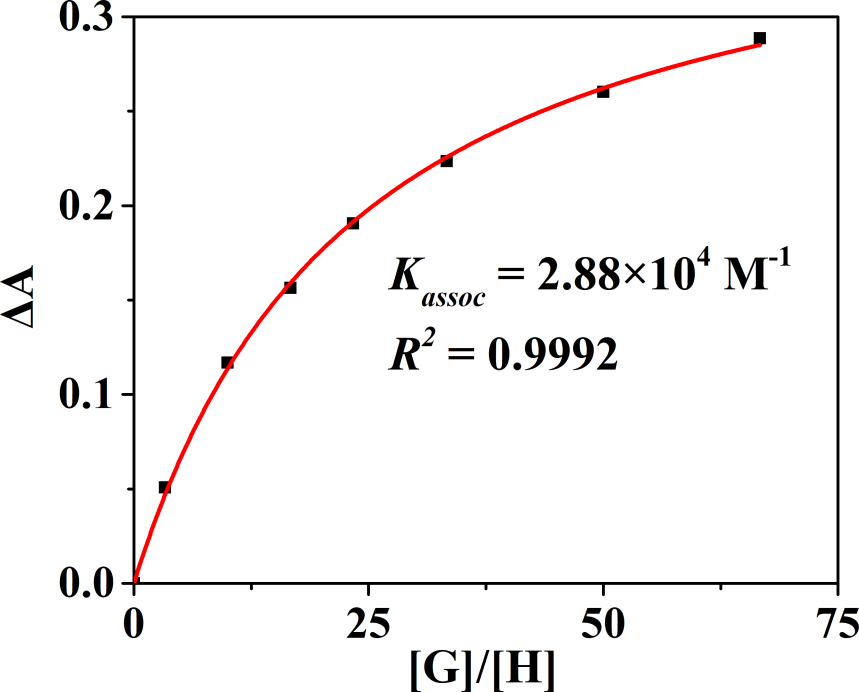


**Figure S4.** Changes in *ΔA* at 420 nm for evaluating *K*_assoc_, the solid line represents the non-liner least square fit for 1:1 complexation. [(*R*)-**H**] = 1.5 × 10^−6^ M; [(*R*)-PPDA]/[(*R*)-**H**] = 0−67.

**Fluorescence titration.**

Benesi-Hildebrand equations for 1:1 complexes:

$$\frac{I_{0}}{I-I_{0}}= \frac{b}{a-b}\{\frac{1}{K\left[ G \right]}+1\}$$

Where I_0_ is the inherent fluorescence intensity of the porphyrin dimer host, I is the fluorescence intensity in the presence of guest, [G] is the guest concentration, and K is the association constant, a and b are constants. The association constants K were obtained from the ratio of the y-intercept to the slope of the plots.^[S3]^


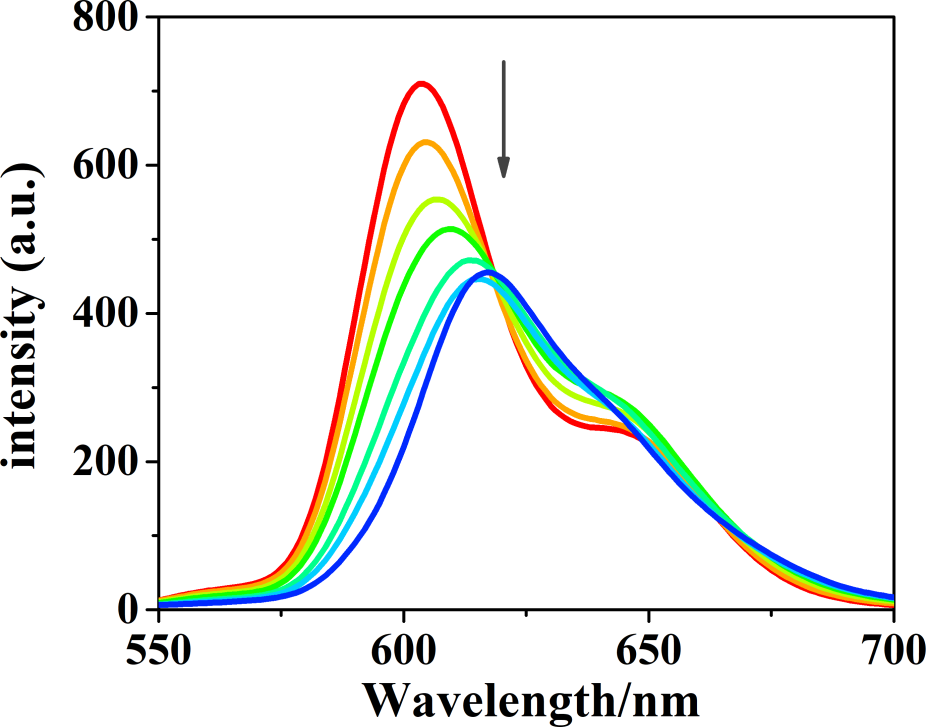


**Figure S5.** The fluorescence spectral change upon titration of (*R*)-**H** with (*S*)-PPDA in CHCl_3_ at 298 K, [(*R*)-**H**] = 1.5 × 10^−6^ M; [(*R*)-PPDA]/[(*R*)-**H**] = 0−67, *λ_Ex_* = 415 nm.


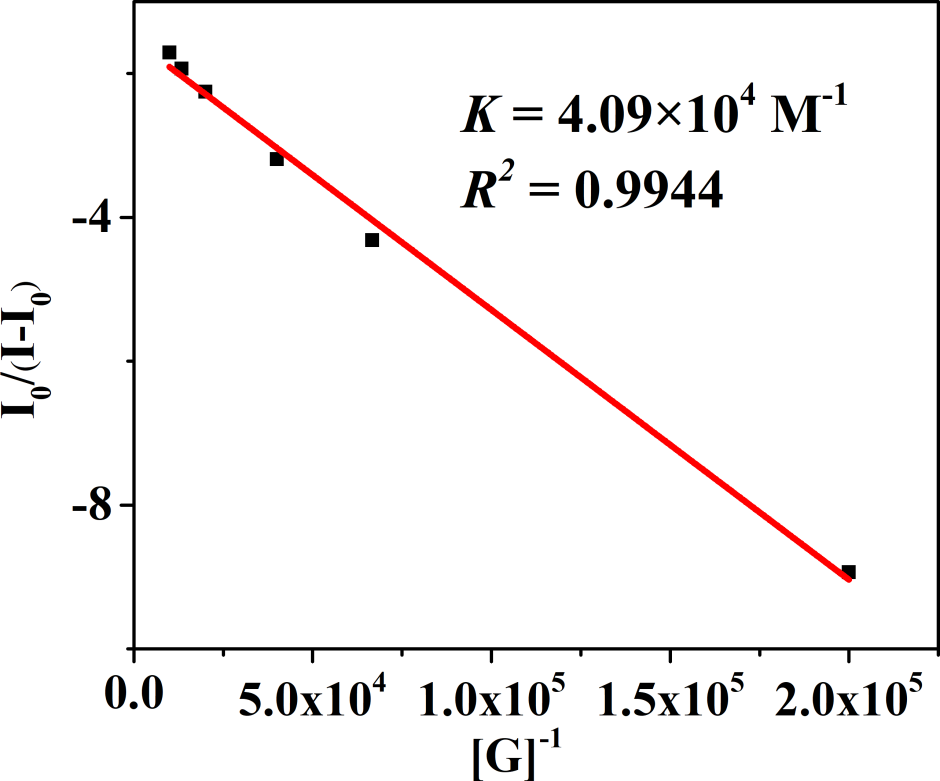


**Figure S6.** The Benesi-Hildebrand plot upon titration of (*R*)-**H** with (*S*)-DACH in CHCl_3_ at 298 K. [(*R*)-**H**] = 1.5 × 10^−6^ M; [(*R*)-PPDA]/[(*R*)-**H**] = 0−67, *λ_Ex_* = 415 nm, monitored at 604 nm.


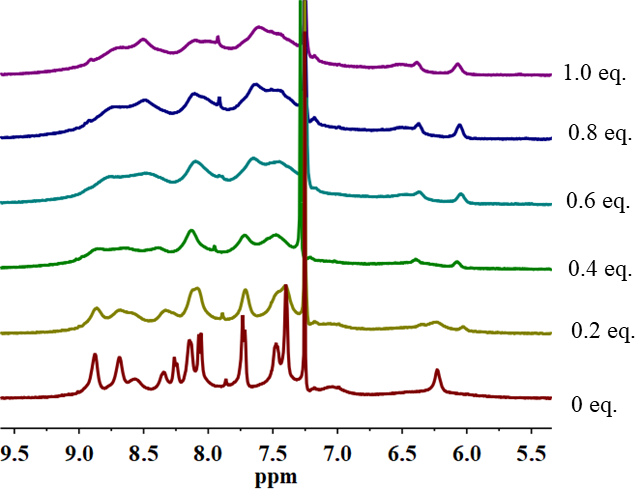


**Figure S7.** Partial ^1^H NMR titration spectra of (*S*)-**H** (0.75 mM) with (*S*)-PPDA (0.0-1.0 equiv, 0.2 equiv additions) at 298 K in CDCl_3_.

**Computational details**: DFT calculations on the optimization of geometric molecular structures were performed at the B97D/6-31G(D) level using Gaussian 09 (*Version D.01*) program. ^[S4]^ The torsion angle (*Φ*) between the two chromophores is the spatial angel of C15-C5-C5′-C15′. The interchromophoric distance is the distance of Zn-Zn.

**References:**

[S1] Zhang, X., Li, Y., Qi, D., Jiang, J., Yan, X., & Bian, Y. (2010). Linkage Dependence of Intramolecular Fluorescence Quenching Process in Porphyrin-Appended Mixed (Phthalocyaninato)(Porphyrinato) Yttrium(III) Double-Decker Complexes. *Journal of Physical Chemistry B,* 114, 13143-13151. doi: 10.1021/jp106020t

[S2] Thordarson, P. (2011). Determining association constants from titration experiments in supramolecular chemistry. *Chemical Society Reviews,* 40, 5922-5923. doi**:** 10.1039/c0cs00062k

[S3] Jiao, J., Tan, C., Li, Z., Liu, Y., Han, X., & Cui, Y. (2018). Design and Assembly of Chiral Coordination Cages for Asymmetric Sequential Reactions. *Journal of the American Chemical Society,* 140, 2251-2259. doi: 10.1021/jacs.7b11679

[S4] Frisch M., Trucks G., Schlegel H., Scuseria G., Robb M., Cheeseman J., et al. Gaussian 09, Revision D.01. Wallingford, CT: Gaussian Inc.; **2013**.
